# Supplementary material for: Instruments for assessing back pain in athletes: A systematic review
Source: PLoS One. 2023 Nov 3;18(11):e0293333. doi: 10.1371/journal.pone.0293333 (PMC10624266; doi:10.1371/journal.pone.0293333)
Supplement: S1 Annex — (DOCX) [file pone.0293333.s001.docx]

| **Annex 1-** Search strategies used by the respective database | |
| --- | --- |
| Database | Search strategies |
| PUBMED | ((pain measurement[MeSH Terms] OR questionnaire[Title/Abstract] OR questionnaires[Title/Abstract] OR instrument[Title/Abstract] OR instruments[Title/Abstract] OR form[Title/Abstract] OR forms[Title/Abstract] OR assessment[Title/Abstract] OR assessments[Title/Abstract] OR score[Title/Abstract] OR scores[Title/Abstract] OR measurement[Title/Abstract] OR measurements[Title/Abstract] OR scale[Title/Abstract] OR scales[Title/Abstract] OR tool[Title/Abstract] OR tools[Title/Abstract]) AND (back pain[MeSH Terms] OR low back pain[MeSH Terms] OR lumbago[Title/Abstract] OR neck pain[MeSH Terms] OR "back pain"[Title/Abstract] OR "low back pain"[Title/Abstract] OR backache[Title/Abstract] OR backaches[Title/Abstract] OR "back ache"[Title/Abstract] OR "back aches"[Title/Abstract] OR "spinal pain"[Title/Abstract] OR "neck ache"[Title/Abstract] OR "neck pain"[Title/Abstract]) AND (athlete[MeSH Terms] OR sports[MeSH Terms] OR athlete[Title/Abstract] OR athletes[Title/Abstract] OR sports[Title/Abstract] OR sport[Title/Abstract] OR sportsman[Title/Abstract] OR sportsmen[Title/Abstract] OR sportswoman[Title/Abstract] OR sportswomen[Title/Abstract]) |
| EMBASE | (questionnaire:ab,ti OR questionnaires:ab,ti OR instrument:ab,ti OR instruments:ab,ti OR form:ab,ti OR forms:ab,ti OR assessment:ab,ti OR assessments:ab,ti OR score:ab,ti OR scores:ab,ti OR scale:ab,ti OR scales:ab,ti OR measurement:ab,ti OR measurements:ab,ti OR tool:ab,ti OR tools:ab,ti) AND (lumbago:ab,ti OR 'back pain':ab,ti OR 'low back pain':ab,ti OR backache:ab,ti OR 'back aches':ab,ti OR backaches:ab,ti OR 'back ache':ab,ti OR 'spinal pain':ab,ti OR 'neck ache':ab,ti OR 'neck pain':ab,ti) AND (athletes:ab,ti OR athlete:ab,ti OR sports:ab,ti OR sport:ab,ti OR sportsman:ab,ti OR sportsmen:ab,ti OR sportswoman:ab,ti OR sportswomen:ab,ti) |
| SCOPUS | (TITLE-ABS ( athlete OR athletes OR sport OR sports OR sportsman OR sportsmen OR sportswoman OR sportswomen)) AND (TITLE-ABS ("back pain" OR "low back pain" OR backache OR backaches OR "back ache" OR lumbago OR "back aches" OR "spinal pain" OR "neck ache" OR "neck pain")) AND (TITLE-ABS (questionnaire OR questionnaires OR instrument OR instruments OR form OR forms OR assessment OR assessments OR score OR scores OR scale OR scales OR measurement OR measurements OR tool OR tools)) |
| CINAHL | TI ( pain measurement OR tool OR tools OR scale OR scales OR measurement OR measurements OR score OR scores OR assessment OR assessments OR form OR forms OR questionnaire OR questionnaires OR instrument OR instruments OR ) AND TI ( back pain OR low back pain OR lumbago OR neck pain OR backache OR backaches OR spinal pain OR neck ache OR neck pain ) AND TI ( athlete OR sports OR athlete OR athletes OR sports OR sport OR sportsman OR sportsmen OR sportswoman OR sportswomen ) OR AB ( pain measurement OR tool OR tools OR scale OR scales OR measurement OR measurements OR score OR scores OR assessment OR assessments OR form OR forms OR questionnaire OR questionnaires OR instrument OR instruments ) AND AB ( back pain OR low back pain OR lumbago OR neck pain OR backache OR backaches OR spinal pain OR neck ache OR neck pain ) AND AB ( athlete OR sports OR athlete OR athletes OR sports OR sport OR sportsman OR sportsmen OR sportswoman OR sportswomen ) |
| SPORT DISCUS | TI ( pain measurement OR tool OR tools OR scale OR scales OR measurement OR measurements OR score OR scores OR assessment OR assessments OR form OR forms OR questionnaire OR questionnaires OR instrument OR instruments OR ) AND TI ( back pain OR low back pain OR lumbago OR neck pain OR backache OR backaches OR spinal pain OR neck ache OR neck pain ) AND TI ( athlete OR sports OR athlete OR athletes OR sports OR sport OR sportsman OR sportsmen OR sportswoman OR sportswomen ) OR AB ( pain measurement OR tool OR tools OR scale OR scales OR measurement OR measurements OR score OR scores OR assessment OR assessments OR form OR forms OR questionnaire OR questionnaires OR instrument OR instruments ) AND AB ( back pain OR low back pain OR lumbago OR neck pain OR backache OR backaches OR spinal pain OR neck ache OR neck pain ) AND AB ( athlete OR sports OR athlete OR athletes OR sports OR sport OR sportsman OR sportsmen OR sportswoman OR sportswomen ) |
