# Supplementary material for: Instruments for assessing back pain in athletes: A systematic review
Source: PLoS One. 2023 Nov 3;18(11):e0293333. doi: 10.1371/journal.pone.0293333 (PMC10624266; doi:10.1371/journal.pone.0293333)
Supplement: S1 Checklist — (DOCX) [file pone.0293333.s002.docx]

| **Section and Topic** | **Item #** | **Checklist item** | **Location where item is reported** |
| --- | --- | --- | --- |
| **TITLE** | | |  |
| Title | 1 | Instruments for assessing back pain in athletes: a systematic review | 1 |
| **ABSTRACT** | | |  |
| Abstract | 2 | Back and neck pain in athletes varies with sport, age, and sex, which can impair athletic performance, thereby contributing to retirement. Studies on back pain in this population use questionnaires to assess components, such as pain intensity and location and factors associated with pain, among others. This study aimed to review validated questionnaires that have assessed back pain in athletes. This systematic review was conducted according to Preferred Reporting Items for Systematic Reviews and Meta-Analyses (PRISMA) by searching the databases Embase, MEDLINE, SPORTDiscus, CINAHL, and Scopus. The articles were selected regardless of language and date of publication. Titles and abstracts were independently selected by two reviewers; disagreements were resolved by a third reviewer. All the steps were conducted using the software Rayyan. The methodological quality of the questionnaire validation articles was assessed using a critical appraisal tool checklist proposed by Brink and Louw. The search returned 4748 articles, of which 60 were selected for this review, including 5 questionnaire validation studies. These articles were published between 2004 and 2022, which were performed in more than 20 countries, particularly Germany (14) and Sweden (5). Thirteen different instruments were identified, of which 46.1% were developed in Europe. The most commonly used questionnaires were the Oswestry Disability Index and Nordic Standardized Questionnaire. In addition, five questionnaire validation studies were selected for methodological quality assessment, with only two studies demonstrating high methodological quality. The following three instruments were identified for assessing back pain specifically in athletes: Micheli Functional Scale, Persian Functional Rating Index, and Athlete Disability Index. This review confirmed that all three instruments were specifically designed to assess this condition. | 1 |
| **INTRODUCTION** | | |  |
| Rationale | 3 | Many questionnaires assessing BP are prevalent in different populations (2, 33, 34). However, athletes are subject to more intense and sport-specific variables, which are not included in these questionnaires (35). Therefore, certain questionnaires have limitations; however, efforts have been made to develop specific questionnaires for athletes (10, 36-40). However, despite these efforts, no instrument is considered the gold standard for athletes (13). | 2 |
| Objectives | 4 | This study aimed to review validated questionnaires that have assessed back pain in athletes. | 1 |
| **METHODS** | | |  |
| Eligibility criteria | 5 | This review included articles whose instruments met the following criteria: a) mentioned prevalence, incidence, intensity, location, functional disability, or other BP-related components, b) assessed athletes and sports-related variables, and c) could be created, adapted, or translated but were validated or at least tested for their reliability. In this research, validated questionnaires were analyzed regardless of the country. Therefore, the same questionnaire could be validated for athletes in different countries and may thus vary based on cultural adaptation.  Thus, the articles that met the following criteria were excluded from this review: a) systematic reviews, reports, opinion articles, response letters, and book chapters, b) research that included individuals with physical or mental disabilities, pregnant or lactating women, and participants with spinal fractures or who have recently undergone surgery, c) studies conducted in specific and/or traditional communities (for example, rural communities, indigenous populations, refugees, uncontacted individuals, and remote and isolated communities). | 4 |
| Information sources | 6 | Embase, MEDLINE, SPORTDiscus, CINAHL, and Scopus. | 3 |
| Search strategy | 7 | (#1) “Pain measurement” OR “Questionnaire” OR “instrument” OR “form” OR “assessment” OR “score” OR “measurement” OR “scale” OR “tool”  (#2) “Back pain” OR “low back pain” OR lumbago OR “neck pain” OR backache OR “spinal pain” OR “neck ache” OR “neck pain”  (#3) “Athlete” OR “sport” OR “sportsman” OR “sportswoman”  (#1) AND (#2) AND (#3) | 4 |
| Selection process | 8 | The results from the search strategies were imported into the Mendeley software; duplicate articles were identified and removed. The first selection phase consisted of reading the title and abstracts to assess whether each article met the eligibility criteria. After this phase, the articles were read completely to confirm their eligibility. All the steps were performed using the Rayyan software, which is specifically designed for conducting systematic reviews (45).  The article-selection step was independently performed by two reviewers, and disagreements were resolved by a third reviewer. Interrater reliability for individual component ratings was determined by calculating the percent agreement and Cohen’s Kappa coefficient. Thereafter, eligible articles were included in this systematic review. | 4-5 |
| Data collection process | 9 | The first selection phase consisted of reading the title and abstracts to assess whether each article met the eligibility criteria. After this phase, the articles were read completely to confirm their eligibility. | 4 |
| Data items | 10a | The following data were extracted from the selected articles: author and year of publication, country of research, participants’ age, name of the evaluation instruments, sport, sample size, level of physical activity, and BP definition. The following data were extracted from the selected instruments: type, name, number of items of the instrument, assessment method and objective of the assessment instrument. The following data were extracted from the studies selected for methodological quality assessment: study (author and year), instrument objective, name (instrument abbreviation), sport/activities assessed by the instrument, validity, internal consistency and reliability. | 5 |
|  | 10b |  |  |
| Study risk of bias assessment | 11 | The methodological quality of the articles that evaluated the measurement properties of the instruments specifically developed for athletes was determined using the Critical Appraisal Tool (CAT) proposed by Brink and Louw (46). The scale consists of 13 items, of which five refer to both validity and reliability, four to validity, and four to reliability studies. Each item is scored as “Yes,” “No,” or “Not Applicable (N/A).” This scale was used by the same independent reviewers. A study is considered of high methodological quality when the score ≥ 60% (47, 48). | 5 |
| Effect measures | 12 | Not applied. | - |
| Synthesis methods | 13a | The article-selection step was independently performed by two reviewers, and disagreements were resolved by a third reviewer | 5 |
|  | 13b | Table 1, 2 and 3. Box 2 and Figure 3. | 9/18 |
|  | 13c | Table 1, 2 and 3. Box 2 and Figure 3. | 9/18 |
|  | 13d | Not applied. | - |
|  | 13e | Not applied. | - |
|  | 13f | Not applied. | - |
| Reporting bias assessment | 14 | The methodological quality of the articles that evaluated the measurement properties of the instruments specifically developed for athletes was determined using the Critical Appraisal Tool (CAT) proposed by Brink and Louw (46). | 5 |
| Certainty assessment | 15 | Not applied. | - |
| **RESULTS** | | |  |
| Study selection | 16a | Preferred Reporting Items for Systematic Reviews and Meta-Analyses flow diagram for study selection. | 7 |
|  | 16b | This review included articles whose instruments met the following criteria: a) mentioned prevalence, incidence, intensity, location, functional disability, or other BP-related components, b) assessed athletes and sports-related variables, and c) could be created, adapted, or translated but were validated or at least tested for their reliability. In this research, validated questionnaires were analyzed regardless of the country. Therefore, the same questionnaire could be validated for athletes in different countries and may thus vary based on cultural adaptation. | 4 |
| Study characteristics | 17 | Table 1 and Table 2. | 9/13 |
| Risk of bias in studies | 18 | Table 3 | 18 |
| Results of individual studies | 19 | Table 1 and Table 2. | 9/13 |
| Results of syntheses | 20a | Table 1, 2 and 3. Box 2 and Figure 3. | 9/18 |
|  | 20b | Not applied. | - |
|  | 20c | Not applied. | - |
|  | 20d | Not applied. | - |
| Reporting biases | 21 | Not applied. | - |
| Certainty of evidence | 22 | Not applied. | - |
| **DISCUSSION** | | |  |
| Discussion | 23a | Some systematic reviews have previously investigated the existence of instruments for evaluating BP (116-118). Systematic reviews organize data objectively and may report significant results that potentially contribute to various studies. Therefore, reviews that systematize instruments for assessing pain in specific populations are relevant because these results may aid researchers in better selection of instruments according to their research objective.  To the best of our knowledge, this review is the first to summarize instruments for assessing BP in athletes. Therefore, the main purpose of this study was to identify the instruments used in the literature to assess BP in athletes and summarize the articles that developed questionnaires for athletes. We hypothesized that few instruments are valid to assess BP in athletes and that the available instruments contribute little to the specific assessment of BP in this population. | 20 |
|  | 23b |  |  |
|  | 23c |  |  |
|  | 23d |  |  |
| **OTHER INFORMATION** | | |  |
| Registration and protocol | 24a | To support this review, we performed and published an article on a systematic review protocol entitled: “Evaluating Instruments for Assessing Back Pain in Athletes: A Systematic Review Protocol” (35). and it was registered in the PROSPERO database (CRD42020201299). | 3 |
|  | 24b |  |  |
|  | 24c |  |  |
| Support | 25 | This research did not receive any specific grant from funding agencies in the public, commercial, or not-for-profit sectors.  The authors would like to show their gratitude to Instituto Federal Goiano, Universidade Federal de Goiás and the Research Group on Child and Adolescent Health (www.gpsaca.com.br (accessed on 30 September 2022)) for the support. | 22 |
| Competing interests | 26 | The authors report no declarations of interest. | 22 |
| Availability of data, code and other materials | 27 | By e-mail the authors (regina.silva@ifg.edu.br) | - |

*From:*  Page MJ, McKenzie JE, Bossuyt PM, Boutron I, Hoffmann TC, Mulrow CD, et al. The PRISMA 2020 statement: an updated guideline for reporting systematic reviews. BMJ 2021;372:n71. doi: 10.1136/bmj.n71

For more information, visit: <http://www.prisma-statement.org/>
