# Supplementary material for: Instruments for assessing back pain in athletes: A systematic review
Source: PLoS One. 2023 Nov 3;18(11):e0293333. doi: 10.1371/journal.pone.0293333 (PMC10624266; doi:10.1371/journal.pone.0293333)
Supplement: Attachment — Submitted filename: comments to authors.docx [file pone.0293333.s003.docx]

Dear authors,

I am glad to have had the opportunity to review this study. Congratulations, it's a nice study with clinical outputs. I think that your research will contribute to the literature. I hope the comments contribute to your study. It will look better with some corrections. Study needs corrections and the authors should carefully address the following issues:

- The article type was entered incorrectly, please correct it.
- Pay attention to punctuation marks and spaces between characters.
- Replace informal uses (we, our, etc.).
- Annex or appendix? Prefer single use.
- There is no appendix 2.

**Abstract**

- Generally acceptable.

**Introduction**

- Generally acceptable.

**Materials and Methods**

- 2.6 Reviewer training: “Lastly, the training process…” Detail or reference this process.
- Spelling errors can be seen throughout the text. I will mention some of it, but it should be reviewed on this.

**Results**

- Page 12, line 180: Pay attention to dots and parentheses.
- In Table 2, the reliability of reference 37 is given as Cronbach's alpha, check it.
- Page 24: Delete the extra comma in the sentence above Table 3.

**Discussion:**

- Generally acceptable.
- Also in the last paragraph, can you tell which instruments you would recommend using for pain or disability based on these results?

**Abstract:**

- GRADE should be clearly written in the Results section.
- The studies are largely high-risk and the level of evidence is low, please rewrite the Conclusions accordingly.

**Introduction**

- Generally acceptable.
- The purpose of the systematic review and meta-analysis should be explained. Which parameters created your outcomes? Mention these.

**Methods**

- Page 7, line 13: (https://www.crd.york.ac.uk/ prospero/) This link is not accessible.
- Page 7, line 43: “There were no limitations in terms of population gender, , study settings, date, and publication language.” Delete the comma.
- Page 10, line 26: “In this review, the GRADE system was used to rate the overall quality of the evidence of the included studies based on the outcomes of interest (38).” It is mentioned here for the first time in the text. Please write clearly.

**Results**

- Page 11, line 45: “Another seven studies were included through manual search (93–98).” Seven studies were said to be included, but only 6 references. Missing a reference?
- Page 11, line 45: “In total, 48 studies published from 1986 to 2022 were included in this review.” Could it be 52? This number is also written on the diagram.
- Page 12, line 38: “Overall, 45 studies (86.5%) were considered to have a high risk of bias, and the subsequent seven studies (56,80,81,84–86,96) (13.5%) have "some concerns" for the overall RoB. The risk of bias assessment for all included studies is presented in **Supplement F (1)**.” The abbreviation (RoB) must appear where it is first used.
- Page 13, line 33: “**Effect of Acupuncture based on outcomes and comparisons.”** Remove the dot. The "a" of Acupuncture should be small.
- Page 13, line 49: “Fifteen studies (51,60,61,66,67,70,80,81,84,86,87,93,95–97) reported pain intensity for this comparison for both myogenous and mixed TMDs.” What does mixed TMDs mean? Which TMDs diagnose? Mixed TMDs are included in many parts of the text. However, which diagnoses were evaluated as mixed TMDs in which study? Myogenic+arthrogenic or myogenic+discogenic? Please try to include this in the tables. this in the tables.
- Page 14, line 45: “Seven studies in this TMDs subgroup evaluated the effect of traditional acupuncture vs. sham or non-active treatment (67,70,80,87,95–97)” There should be a dot at the end of the sentence.
- Page 20, line 10: “2. Laser acupuncture vs lower laser therapy” Correct it as low level laser therapy. Also correct any usage in the text.
- Page 24, line 29: “Two studies study looked at the maximal mouth opening when comparing laser acupuncture vs. lower laser therapy.” Correct the spelling mistakes.
- How did you decide on the I^2^ reference intervals and how did you interpret the heterogeneities?

**Discussion:**

- Page 27, line 13: “One study by Branco et al., (65), …” Use the comma in the right place.
- The discussion section was like a description of the results and a summary of the included studies. How do you think these results can be interpreted, it would be better if you talk about them from a clinical point of view.

**Tables:**

- Page 60: From reference 9 to reference 32, the table is intertwined and cannot be read. Make the necessary corrections.
- Page 91, line 23: Correct the spelling of "CRITICAL".
