# Supplementary material for: Instruments for assessing back pain in athletes: A systematic review
Source: PLoS One. 2023 Nov 3;18(11):e0293333. doi: 10.1371/journal.pone.0293333 (PMC10624266; doi:10.1371/journal.pone.0293333)
Supplement: Attachment — Submitted filename: Letter reviewer #2 12 06.docx [file pone.0293333.s005.docx]

Reviewer #2

We would like to express our thanks to the editor and two reviewers for their thoughtful and in-depth comments concerning our manuscript. Your suggestions helped us improve the quality of our paper. We carefully considered every comment and made all the appropriate changes. Reviewer comments are detailed first, and our responses to the points raised follow (*italics*). We have highlighted the changes made to the body of the manuscript using red coloured text in this document.

**Additional Editor Comments**

1. Overall, I would like to appreciate the authors for their effort to provide this important and interesting work

*Author´s response: Thank you very much for your positive feedback.*

2. This manuscript titled "Instruments for assessing back pain in athletes: a systematic review". Accordingly, the authors should focus on the study question "Back pain in athletes". However, the authors started abstract with back and neck pain.

*Author´s response: Thanks for your careful review. We agree with the reviewer. We removed the word as suggested, as follow:*

“Back pain in athletes varies with sport, age, and sex, which can impair athletic performance, thereby contributing to retirement.” (Lines 15-16)

**Introduction**

3. The authors should explain the importance of the study in detail.

*Author´s response: Thanks for your in-depth review. The importance of this study was highlighted in more details, as follow:*

“This study is relevant because the demand in health care, combined with the need to use evaluative instruments, has contributed to increase, for example, the pressure on health care professionals to ensure the implementation of evidence-based practice. Thus, publication of systematic review studies, as well as others that synthesize research results, is an important action for evidence-based practice. Moreover, this type of study serves to guide the development of projects, indicating new directions for future investigations.” (Lines 76-81)

4. The authors should add the hypothesis of the study.

*Author´s response: Thanks for your careful review. We also include our hypothesis in the introduction, as follow:*

“Therefore, the aim of this study was to review the scientific literature related to validated questionnaires to evaluate BP in athletes. The hypothesis is that there are few valid and reliable instruments to evaluate BP in athletes and that the existing ones contribute little to the specific assessment.” (Lines 81-84).

**Discussion**

5. The strengths of this review should be demonstrated with detailed explanation.

*Author's Response: Thanks for your in-depth review. The strengths of this review are demonstrated, as follows:*

“Considered as strengths of this review the fact that this article is the first that summarizes instruments that assess BP specifically for athletes. Furthermore, this review selected articles in all languages and without fixing a publication date, and these criteria are relevant because they do not limit the ability to identify articles in the literature. Lastly, the search strategy was conducted in five recognized databases (Embase, MEDLINE, SPORTDiscus, CINAHL, and Scopus).” (Lines 389-394).

**Conclusion**

6. Future directions and recommendations need to be clarified.

*Author´s response: Thanks for your careful review. Message to readers and researchers was reinforced, as follows:*

“To future readers and researchers in the area, we suggest new research articles and instruments, because the effectiveness of treatment and its follow-up depend on a reliable and valid pain assessment and measurement.” (Lines 431-434)
