# Supplementary material for: Instruments for assessing back pain in athletes: A systematic review
Source: PLoS One. 2023 Nov 3;18(11):e0293333. doi: 10.1371/journal.pone.0293333 (PMC10624266; doi:10.1371/journal.pone.0293333)
Supplement: Attachment — Submitted filename: Resposta ao revisor 28 08 2023.docx [file pone.0293333.s006.docx]

Reviewer #1

Response to editor and reviewers

We would like to express our thanks to the editor and two reviewers for their thoughtful and in-depth comments concerning our manuscript. Your suggestions helped us improve the quality of our paper. We carefully considered every comment and made all the appropriate changes. Reviewer comments are detailed first, and our responses to the points raised follow (*italics*). We have highlighted the changes made to the body of the manuscript using red coloured text in this document

**Additional Editor Comments**

Please review your reference list to ensure that it is complete and correct. If you have cited papers that have been retracted, please include the rationale for doing so in the manuscript text, or remove these references and replace them with relevant current references. Any changes to the reference list should be mentioned in the rebuttal letter that accompanies your revised manuscript. If you need to cite a retracted article, indicate the article’s retracted status in the References list and also include a citation and full reference for the retraction notice.

*Author´s response: The reference list was completely checked, and we identified a duplicate in references 80 and 81, so reference 81 was removed and the numbering was completely updated. We also inform you that no retracted reference was used in this manuscript. We also inform you that the manuscript was revised in English by native speaker.*

Reviewer #1:

The requested corrections have been corrected carefully but can be published with a correction of a few minor points. So thank you and congratulations to the authors.

*Author´s response: Thanks for your careful review.*

1. Informal uses still exist in the text. Please correct using the "Find" option one by one.

*Author´s response: Thanks for your careful review.* *Informal usage has been substituted in several passages of the text, as follow:*

*“To support this review, was carried out and published an article on a systematic review…” (Lines 89-90)*

*“Moreover, research has been carried out in other countries, such as in Norway…”(Lines 227-228)*

“This research identified 24 (43.7%) articles using ODI (103) and 14 (25.4%) using SNQ (104).” (line 258)

“Here are some recommendations for creating a new instrument for assessing BP in athletes in the following text.” (line 399)

“…, it is suggested new research articles and instruments, …” (line 433)

2. The Reference 37's reliability stands as Cronbach alpha. Internal consistency is also an indicator of reliability, but this is included in the previous column. If ICC is not available, indicate it.

*Author´s response: Thanks for your careful review.* Can in use the intraclass correlation coefficient (ICC) for test/retest reliability. However, the authors of the text did not use ICC. As reliability can be verified by Cronbach's alpha, we agree with your suggestion to indicate internal consistency as an indicator of reliability. We have therefore made the correction in Table 2.
