# Supplementary material for: Instruments for assessing back pain in athletes: A systematic review
Source: PLoS One. 2023 Nov 3;18(11):e0293333. doi: 10.1371/journal.pone.0293333 (PMC10624266; doi:10.1371/journal.pone.0293333)
Supplement: Attachment — Submitted filename: Cover Letter FINAL.docx [file pone.0293333.s007.docx]

PLOS ONE Decision: Revision required [PONE-D-23-08713R2] - [EMID:96f974367d29e64a]

Instruments for assessing back pain in athletes: a systematic review

PLOS ONE

Dear Dr. Ferreira Silva,

Thank you for submitting your manuscript to PLOS ONE. After careful consideration, we feel that it has merit but does not fully meet PLOS ONE’s publication criteria as it currently stands. Therefore, we invite you to submit a revised version of the manuscript that addresses the points raised during the review process.

We would like to express our thanks to the editor and two reviewers for their thoughtful and in-depth comments concerning our manuscript. Your suggestions helped us improve the quality of our paper. We carefully considered every comment and made all the appropriate changes. Reviewer comments are detailed first, and our responses to the points raised follow (italics). We have highlighted the changes made to the body of the manuscript using red coloured text.

**Additional Editor Comments**

Please review your reference list to ensure that it is complete and correct. If you have cited papers that have been retracted, please include the rationale for doing so in the manuscript text, or remove these references and replace them with relevant current references. Any changes to the reference list should be mentioned in the rebuttal letter that accompanies your revised manuscript. If you need to cite a retracted article, indicate the article’s retracted status in the References list and also include a citation and full reference for the retraction notice.

Author´s response: The reference list was completely checked. We also inform you that no retracted reference was used in this manuscript. We also inform you that the manuscript was revised in English by native speaker.

Reviewer #2:

- Would like to thank the authors for their responses to all previous comments.

Author´s response: Thanks for your careful review

- Discuss the implications and strengths of this review.

Author´s response: Thanks for your careful review. A paragraph has been included which highlights more of the strengths of this review, as follow:

Another strength of this review is that it presents a protocol previously structured by the same group of researchers, which synthesizes the authors’ ideas for carrying out a systematic review. Moreover, this review reduces controversies in the literature, since it does not result from the number of studies in favor of a particular intervention, but form the sum of several case studies, identified and published in specific databases that are widely used in the literature. Finally, another important positive element to be highlighted is the fact that this review directs future studies to different areas of activity, it can be used as a tool to contribute to research in medicine, physiotherapy, sport, among others. (Lines 403-410)

- Explain the limitations in detail.

Author´s response: Thanks for your careful review. Sentences that further highlight the limitations of this review have been included, as follows:

The lack of standardization leads researchers and professionals to use less accurate evaluation strategies. Moreover, the lack of standardization makes it difficult to identify faults and problems, can lead to recurring errors, generates excessive work and rework, and can have low reliability. (Lines 381-384)

To find the right tool for a survey, it is necessary, for example, to decide what to measure and then select the most suitable instrument for measuring the results. The use of tools to improve the way research is carried out has a direct impact on the reliability of the results. (Lines 388- 391)

- Conclusion: add a brief message “………” to readers and researchers

Author´s response: Thanks for your careful review. Periods that further highlight the limitations of this review have been included, as follows:

It is suggested that more precise and reliable tools for assessing pain in this population be developed in the future and that these tools can be used to make the assessment of back pain more precise and standardized. (Lines 450-452)
